# An automatic screening method for strabismus detection based on image processing

**DOI:** 10.1371/journal.pone.0255643

**Published:** 2021-08-03

**Authors:** Xilang Huang, Sang Joon Lee, Chang Zoo Kim, Seon Han Choi

**Affiliations:** 1 Department of Artificial Intelligent Convergence, Pukyong National University, Busan, Korea; 2 Department of Ophthalmology, Kosin University College of Medicine, Busan, Korea; 3 Kosin Innovative Smart Healthcare Research Center, Kosin University Gospel Hospital, Busan, Korea; Cairo University Kasr Alainy Faculty of Medicine, EGYPT

## Abstract

**Purpose:**

This study aims to provide an automatic strabismus screening method for people who live in remote areas with poor medical accessibility.

**Materials and methods:**

The proposed method first utilizes a pretrained convolutional neural network-based face-detection model and a detector for 68 facial landmarks to extract the eye region for a frontal facial image. Second, Otsu’s binarization and the HSV color model are applied to the image to eliminate the influence of eyelashes and canthi. Then, the method samples all of the pixel points on the limbus and applies the least square method to obtain the coordinate of the pupil center. Lastly, we calculated the distances from the pupil center to the medial and lateral canthus to measure the deviation of the positional similarity of two eyes for strabismus screening.

**Result:**

We used a total of 60 frontal facial images (30 strabismus images, 30 normal images) to validate the proposed method. The average value of the iris positional similarity of normal images was smaller than one of the strabismus images via the method (*p*-value<0.001). The sample mean and sample standard deviation of the positional similarity of the normal and strabismus images were 1.073 ± 0.014 and 0.039, as well as 1.924 ± 0.169 and 0.472, respectively.

**Conclusion:**

The experimental results of 60 images show that the proposed method is a promising automatic strabismus screening method for people living in remote areas with poor medical accessibility.

## Introduction

Strabismus, or “crossed-eyes,” is one of the most common ocular diseases wherein the eyes do not align with each other when focusing on an object [1]. In the USA, it has been reported that strabismus affects approximately 4% of the population and commonly occurs in children [2, 3]. Strabismus has a serious impact on human life. It is the main cause of amblyopia, which leads to irreversible permanent vision loss [4, 5]. In addition, a patient who has had traumatic brain injuries or a stroke can suffer from strabismus [6], and consequently is more likely to experience mobility challenges, depression, and systemic disease [7]. As a result, a timely strabismus screening becomes important and essential for preventing strabismus.

So far, there are multiple ways to complete strabismus screening. Traditional strabismus screening is conducted manually by ophthalmologists through many tests, such as the cover and uncover test, prism cover test, and the Hirschberg test. To reduce the long screening duration of the traditional methods, many digital tools are introduced to tackle the problems. Several authors have suggested utilizing the photoscreener to perform strabismus screening [8–10]. For large communities such as schools, eye trackers [11–13] have been applied to determine the presence of strabismus. Also, virtual reality headsets with pupil-tracking technology have been used to measure the ocular deviation for strabismus screening [14, 15]. However, these methods require additional expenses for advanced equipment, which may be impractical in areas with limited medical resources. More recently, to relieve the labor burden and help experts screen for strabismus in a low-cost way, automatic strabismus screening using digital images has become a popular topic. Almeida et al. [16] identified strabismus in digital images featuring the Hirschberg test. Almeida et al. [17] used images with gaze positions to detect strabismus. Valente et al. [18] achieved strabismus screening through images featuring the cover test. Lu et al. [19] proposed a deep learning method for strabismus detection using a telemedicine dataset. Also, Zheng et al. [20] applied a pretrained deep learning model on the gaze photographs to achieve strabismus screening. Although the deep learning methods have achieved excellent performance, the screening results are difficult to interpret due to their opaque internal learning. In addition, the methods of using digital images taken through traditional tests still lack consideration of situations wherein patients in remote districts may not be available to undergo strabismus screening in hospitals.

To alleviate the difficulty of strabismus screening for people in remote districts and to help ophthalmologists diagnose strabismus faster in a low-cost manner, this work proposes an easy-to-use automatic screening method for strabismus based on image processing. The proposed method uses a frontal facial image from a patient, and it measures the deviation of the positional similarity of two eyes within the image, which aims to provide ophthalmologists with interpretable information for the diagnosis of strabismus. We validated the proposed method by testing the images provided by the Kosin University Gospel Hospital.

## Materials and methods

### Ethics statement

The study adhered to the tenets of the Declaration of Helsinki, and the Institutional Review Board of the Gospel Hospital of Kosin University (KUGH-IRB 2020-03-034) approved the images used in this study. Each participant provided informed written consent after receiving a clear explanation including the purpose of the study and the image usage, and minor participants’ consent was obtained via their parents or guardians. Additionally, participants who suffer strabismus were informed that their participation in the study would not affect the provision of ophthalmic care.

### Face-detection model

Face detection is one of the most challenging issues in image processing, and the successful detection of faces in images is affected by many factors such as illumination, facial features, and occlusion. Despite these difficulties, much progress has been made in face detection, and numerous algorithms have shown remarkable performance in various scenarios. In eye-detection systems [21, 22], face detection is the first step to locating the face position and influences the detection accuracy on the eye region. Therefore, for strabismus screening, which requires precise positioning of the eye region, a stable and accurate face-detection algorithm is essential.

With the rapid development of deep learning, the use of deep learning methods, namely convolutional neural network (CNN), for face detection has received much attention. CNN is a specialized type of neural network model that uses multiple convolutional filters to extract abstract features from raw input images and learns the features by adjusting the filter weights [23, 24]. To perform accurate and reliable face detection for the images taken under various situations, our method employed a CNN-based face detection provided by the Dlib toolkit [25]. It detects faces more accurately than traditional methods, such as a histogram of oriented gradients [26] and scale-invariant feature transform [27], which can be attributed to its learnable feature extractors.

### Facial landmark detector

**Fig 1:**
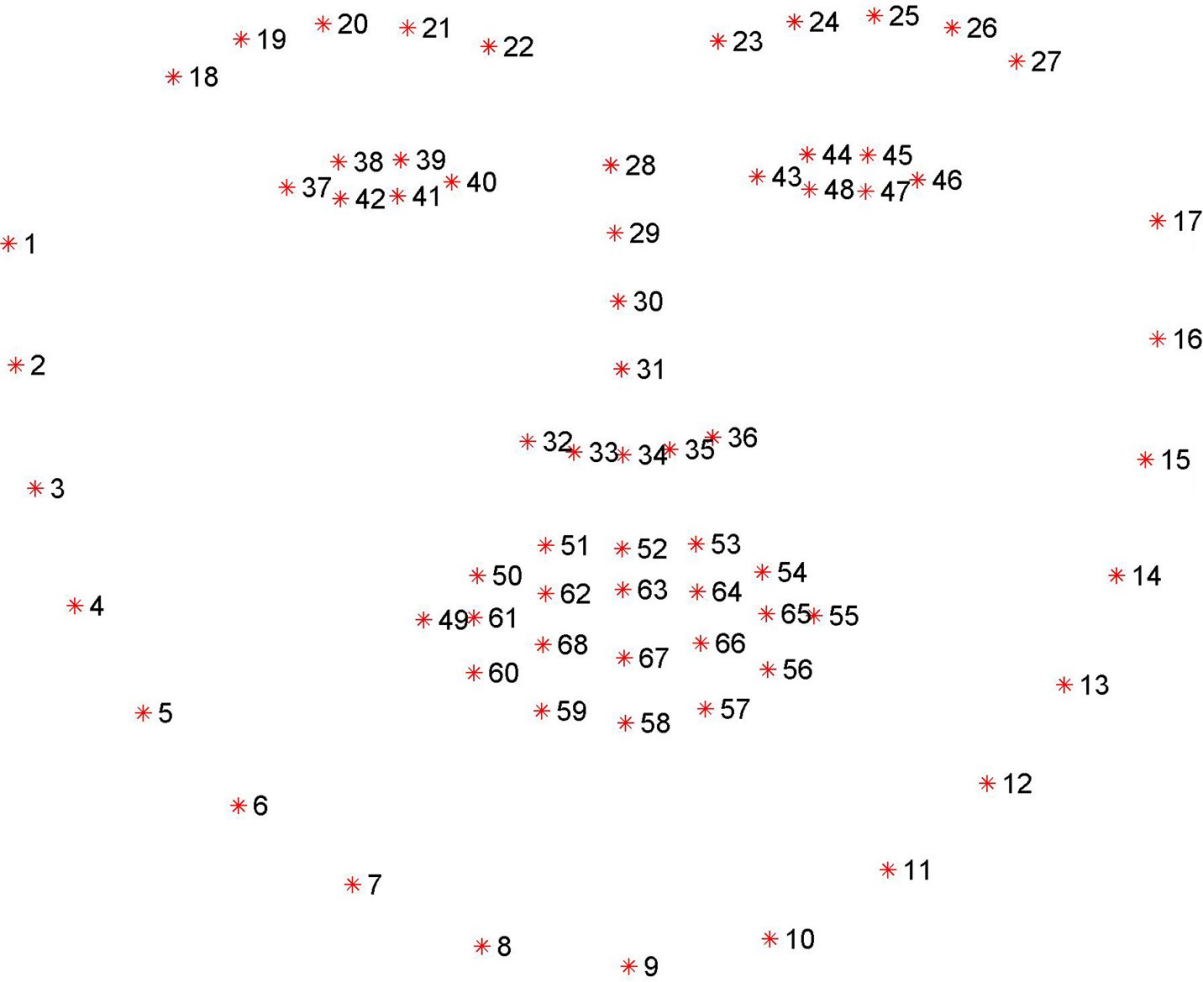
A 68 facial landmark detector pretrained from the iBUG 300-W dataset. Each number represents a predefined feature on the face. For eye extraction, we only considered the index numbers from 37 to 48 on the eye region.

If the face can be detected within the image, the following step is to locate the eye region. To achieve this, we employed a facial landmark detector to locate the eye region based on the position information of the detected face. We developed the detector using an ensemble of regression trees [28], and pretrained it using an iBUG 300-W dataset [29] to extract 68 facial landmarks, as shown in Fig 1. It takes an image containing a facial region as the input and then outputs a set of facial features. All of the feature positions are represented by the index array from 1 to 68. For extracting the eye region, we used the coordinates of the index from 37 to 46 to create a region wherein the two eyes are included.

### Otsu’s binarization

In image processing, color or grayscale images are usually converted to binary images so that the target remains in the images while ignoring backgrounds that we are not concerned with. For this reason, an optimal threshold value must be manually specified to distinguish the target from the irrelevant object, which depends on the experience of the designer. However, due to the fact that illumination may be different in each image, manually selecting the threshold value for the images is tedious and infeasible. To tackle this problem, our method adopts Otsu’s binarization [30], which automatically determines the optimal threshold value by searching the grayscale level that minimizes intraclass intensity variance. In this study, we used Otsu’s binarization to eliminate the influences of the skin color and the sclera (i.e., the white of the eye) on iris positioning.

### HSV color model

In color image processing, the HSV color model is an alternative representation of the RGB color model, where H, S, and V represent hue, saturation, and value (also known as brightness), respectively. The HSV color model is widely used in image classification [31–33] and image segmentation because it can detect targets with specific colors [34, 35]. In this work, the HSV color model aims to eliminate the backgrounds that cannot be eliminated through Otsu’s binarization by setting the upper and lower bound colors of H, S, and V. The HSV color model can be seen as a supplement binarization method to Otsu’s method.

## Procedure

**Fig 2:**
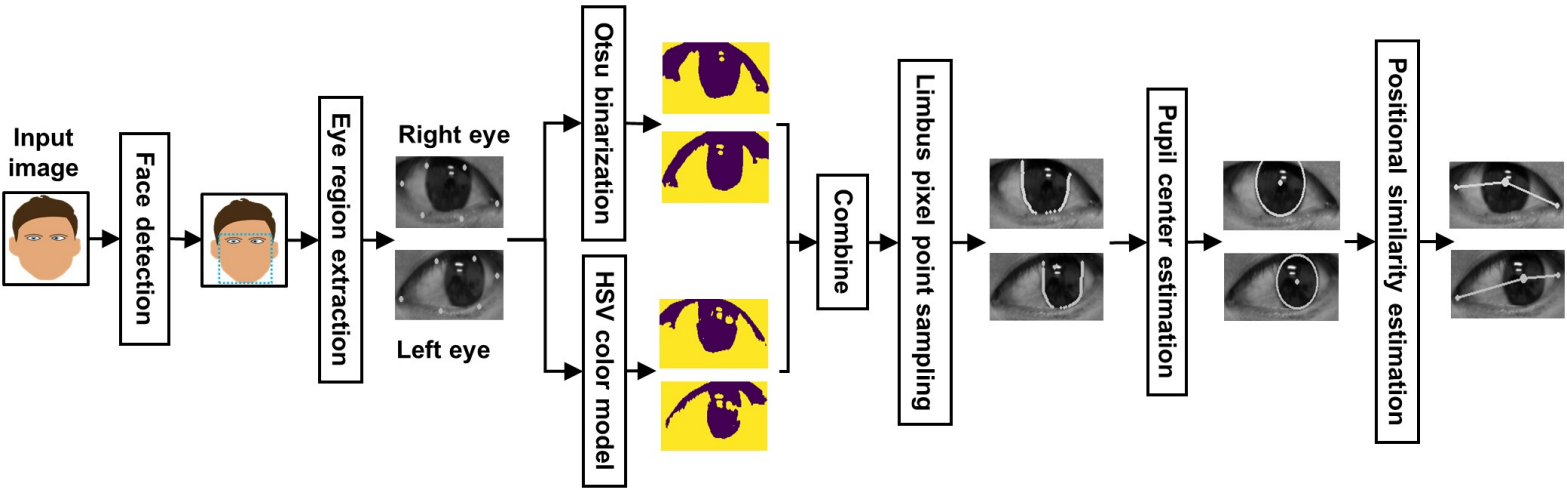
The flowchart of the proposed method. A frontal facial image is sent to the face detection model to identify the face region and the detected face region is subsequently used to extract the eye region through the facial landmark detector. Otsu’s binarization and the HSV color model are applied to the extracted eye region image, and the results from two methods are used to form a new image. The pixel points located at the limbus are sampled and used to estimate the pupil center by the LSM. Finally, the positional similarity of the iris on both eyes is computed for strabismus screening. (Note that eye images displayed in this paper were converted to gray to protect private information. The proposed method actually uses RGB images as the input to perform strabismus screening).

To demonstrate the proposed method, we show the flowchart of the method in Fig 2. The input of the method is an RGB frontal facial image. This image is sent to the CNN-based face detection model to locate the facial region, and the facial landmark detector is applied subsequently to extract the eye region from the facial region. For the eye region image, Otsu’s binarization and the HSV color model are separately applied to remove the background and the results from two thresholding methods are combined to form a new image. With this image, we sampled the pixel points that lie on the limbus and derive the pupil center utilizing the least square method (LSM). The final step is to use the coordinates of the pupil center and medial and lateral canthus to compute the positional similarity of two eyes for strabismus screening.

In the first stage, the proposed method takes an RGB frontal image with a resolution of 1920×1280 as the input, which can be easily obtained through digital devices such as smartphones and cameras. Due to the fact that the use of CNN as the face-detection model may occupy a lot of memory resources, the input image is adjusted to half its size (i.e., 960×640 pixels) before being sent to the model to reduce the computational cost. If a facial region can be detected by the model, it returns the coordinate of a bounding box that encloses the facial part, which is used subsequently for the facial landmark detector to predict the location of 68 facial landmarks that match the facial features.

**Fig 3:**
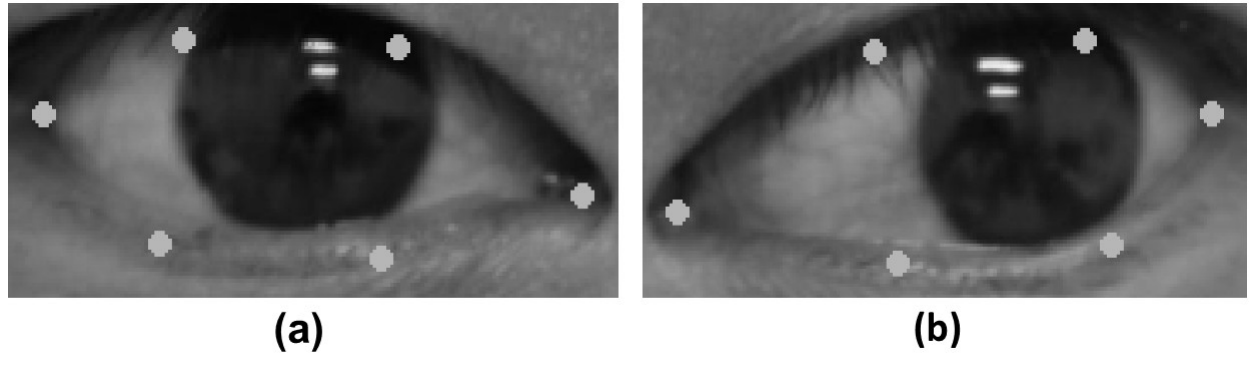
Eye extraction using the 68 facial landmark detector. (a) Result of right eye area extraction. (b) Result of left eye area extraction. Dots represent the landmarks surrounding the eye region.

In the eye-extraction stage, our method attempts to search for the eye region and extract it from the detected facial image. From the facial landmark detector, we observed that the index array of the right eye region ranges from 37 to 42 and 43 to 48 for the left eye region. For extraction of the right eye region, we used the horizontal pixel value of index 37 and the vertical pixel value of index 38 as the start coordinate and the horizontal value of index 40 and the vertical value of 41 as the end coordinate. With these coordinates, the right eye region can be extracted by taking the start coordinate as the upper left corner of the rectangular frame and the end coordinate as the lower right corner. The left eye region can be extracted through similar operations. Nevertheless, the detector may not accurately locate the facial landmarks for the eye region, which affects the extraction of the complete eye region. Considering this issue, we added an extra expansion parameter to increase the size of the rectangular frame. In other words, the parameter simultaneously increases the horizontal and vertical pixel units to ensure that a complete eye region can be included. In this study, we used an expansion parameter of 10-pixel units. An example of the eye region extraction is shown in Fig 3.

**Fig 4:**
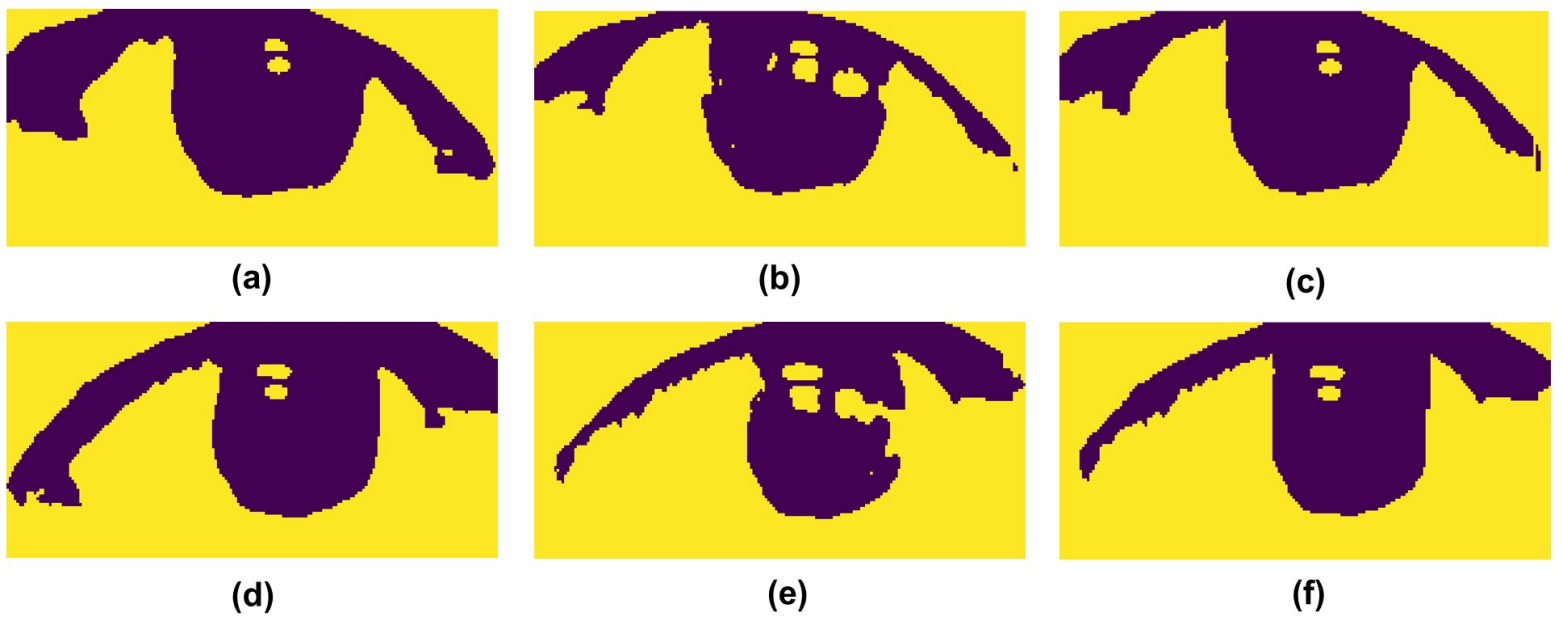
Image processing on eye regions. (a)-(c) show the results of Otsu’s binarization, the HSV color model, and the combination of two methods on the left eye region. (d)-(f) are the results of the right eye region.

The proposed method utilizes Otsu’s binarization and the HSV color model to remove the objects that affect the positioning of the pupil center. First, the eye region images are filtered with a 5x5 Gaussian kernel to remove the noise. Then, the image is converted into grayscale, and Otsu’s binarization is appied to remove the skin and sclera so that only the iris region remains. With Otsu’s binarization, the pixel values smaller than the determined threshold value are set to 0 (black), and the pixel values bigger than the threshold value are set to 255 (white). Despite the fact that Otsu’s binarization can automatically determine a proper threshold value to remove most of the background of the eye region, some black backgrounds (e.g., the shadows generated by the eyelash) remain and affect the result of iris localization. Thus, the proposed method further converts the eye region image to the HSV image via the HSV color model to remove the shadows. To be specific, the HSV color model aims to separate the eye region from the shadows by setting the arrays of the upper and lower bound color to a desired color value. In this work, the lower bound color array was set to [0, 0, 0], which represents the black color. For the upper bound color array, we set it as [180, 255, v], where v is determined by the average of the gray values of the eye region. Although the HSV image may contain less shadow than the binarization image, it is susceptible to the predefined color value. From this point of view, the direct use of the HSV image may cause pixel loss inside the eye region. Therefore, the proposed method combines the binarization and HSV images to form a new image with a separated iris and shadows. To achieve this, the method starts to enumerate the pixel values in each column of the HSV image and stores the first height value with a pixel value of 0 in each column. Then, the binarization image sets the pixel values from the corresponding height value to the maximum height value (bottom) of each column to 255. An example of the image processing on the eye region is shown in Fig 4.

**Fig 5:**
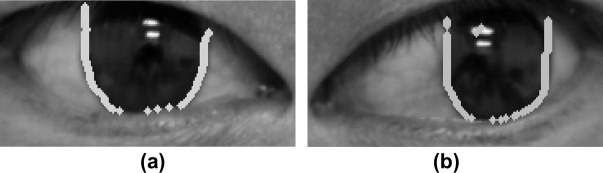
The result of pixel-point sampling on the left and right eye limbus. (a) Right eye region. (b) Left eye region. Dots represent the sampled pixel points on the limbus.

Having the image processed in the previous stage, our method takes a few steps toward finding the coordinates of the pupil center. First, we selected an initial coordinate within the image. Owing to the fact that the iris is usually located at the center part of the image, the center coordinate of the image can be the initial coordinate. If the pixel value of the initial coordinate is 255 (i.e., the initial point is not within the iris region), we utilized the height value of the initial point to search for the iris region along the width of the image until the width value had the pixel value of 0. Second, using the coordinate obtained from the first step, the method searches for the maximum height value of the iris region by comparing the height value of coordinates within the iris. Finally, beginning with the coordinate that has the maximum height value, we sampled all of the pixel points located at the limbus from the bottom to the top of the iris region. Fig 5 visualizes the result of the pixel-point sampling.

**Fig 6:**
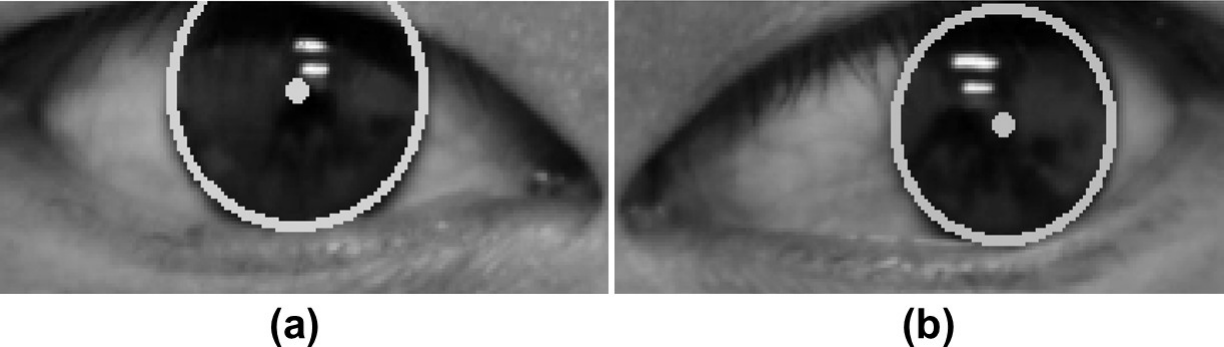
Estimation of pupil center coordinate by the LSM. (a) Right eye region. (b) Left eye region. Dots represent the estimated pupil center.

Using the collected pixel points, our method attempts to determine the center coordinate of the pupil. To this end, we approximated the collected points by employing the LSM, which aims to estimate the coordinate of the pupil center by minimizing the mean square geometric distance from the iris radius to the samples, and it can be defined as follows [36]:

$$min\sum_{i=1}^{n}{\left(\sqrt{{\left(x_{i}-a\right)}^{2}+{\left(y_{i}-b\right)}^{2}}-R\right)}^{2}$$ (1)

where *n* is the total number of pixel points, (*x*_*i*_, *y*_*i*_) is the coordinate of the *ith* point, (*a*, *b*) is the center of the pupil, and *R* is the radius of the iris. Since *R* is not known in advance, it is usually set as the mean of the samples. The result of using the LSM to obtain the center coordinate of the pupil is shown in Fig 6.

**Fig 7:**
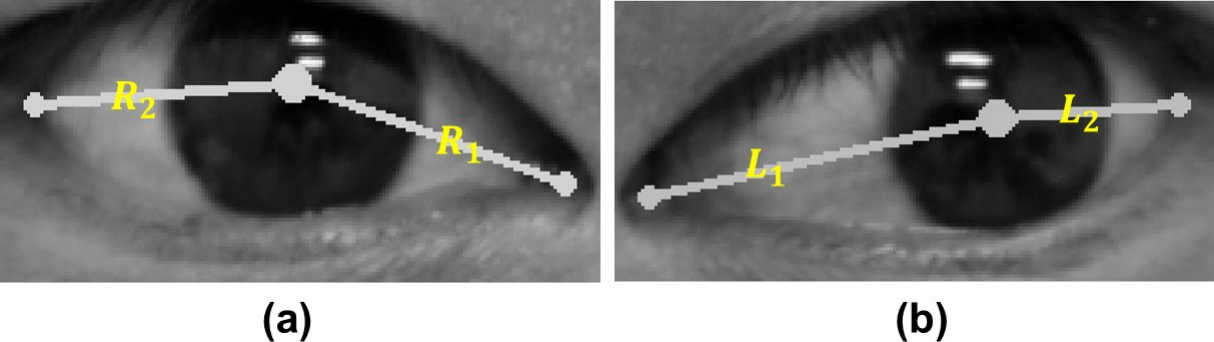
Distance estimation between the pupil center and canthus landmarks. (a) Right eye region. (b) Left eye region. *R*_1_ and *R*_2_ are the distance measurements within the right eye using the estimated pupil center and the coordinates of the medial and lateral canthus. *L*_1_ and *L*_2_ are the distance measurements within the left eye.

To measure the positional similarity of two eyes, we first calculated the distance between the pupil center and the canthus landmarks (e.g., lateral and medial canthus), which are obtained by the facial landmark detector. This distance can be defined as:

$$D=\sqrt{{\left(x_{L}-x_{C}\right)}^{2}+{\left(y_{L}-y_{C}\right)}^{2}}$$ (2)

where (*x*_*L*_, *y*_*L*_) represents the coordinate of the canthus landmarks, and (*x*_*C*_, *y*_*C*_) is the coordinate of the pupil center. Fig 7 visualizes an example of the distance between the pupil center and the landmarks on the eye region.

After calculating the distances, the method utilizes these distances to compute the ratios *R*_1_/*R*_2_, *L*_1_/*L*_2_ for providing the degree of deviation of the iris in each eye. Then, the resulting ratios are further used to measure the positional similarity of the two eyes, which aims to provide the position information for determining the presence of the strabismus. The positional similarity can be defined as:

$$S=\frac{max(\frac{R_{1}}{R_{2}},\frac{L_{1}}{L_{2}})}{mim(\frac{R_{1}}{R_{2}},\frac{L_{1}}{L_{2}})}$$ (3)

The max and min functions are used as the denominator and numerator respectively, to ensure that the value of *S* is always greater than or equal to 1. If *S* is equal or close to 1, the irises are in a similar and symmetrical position with each other, which means the eyes are likely to be normal, otherwise they may be strabismus.

## Statistical analysis

It is noted that the ratios are not normally distributed due to the way in which we calculated the ratios. Therefore, to validate the effectiveness of our method, we applied a nonparametric test, the Mann-Whitney U test, and calculated the *p*-value to show how significant the differences between the ratio of normal and strabismus images are. To this end, we set the null hypothesis (notation *H*_*o*_) and the alternative hypothesis (notation *H*_*A*_) as follows:

$$H_{o}:\mu_{N}\geq \mu_{S},\;H_{A}:\mu_{N}<\mu_{S}$$ (4)

where *μ*_*N*_ and *μ*_*S*_ are the average values of ratios of normal and strabismus images calculated by (3). Under these hypotheses, we aim to verify that the average ratio of normal images is smaller than the one of strabismus images (i.e., *H*_*A*_), which results in demonstrating the effectiveness of the proposed method. If the resulting *p*-value is less than a predefined significance level α (usually 0.05 or 0.001), this indicates there is strong evidence against the null hypothesis *H*_*o*_, and that the alternative hypothesis is accepted. The significance level α was set to 0.001 in this study. We applied the Mann-Whitney U test to the estimated ratios, and the *p*-value = 1.509*10–11 (<0.001) was obtained, indicating the effectiveness of the proposed method.

## Results

We used a total of 60 (30 strabismus images, 30 normal images) frontal facial images taken under various illumination conditions to evaluate the proposed method. In the face- and eye-detection stage, we successfully detected all of the eye regions within the frontal facial images using the CNN-based model and extracted them using the facial landmark detector. The method was implemented using an eight-core AMD Ryzen 7 2700 CPU and was based on Python (version 3.7.9) and OpenCV (version 3.4.2). We conducted the proposed method on the images and calculated the sample mean and standard deviation of the positional similarity estimates of normal and strabismus images, respectively; Table 1 shows the results. The statistical analysis was applied to the estimated values, and the *p*-value = 1.509*10^−11^ (<0.001) was obtained, indicating the effectiveness of the proposed method.

**Table 1:** The sample mean and sample standard deviation of the positional similarity estimates of normal and strabismus images.

	Normal	Strabismus
Sample mean	1.073 ± 0.014	1.924 ± 0.169
Sample standard deviation	0.039	0.472
# of sample	30	30

**Fig 8:**
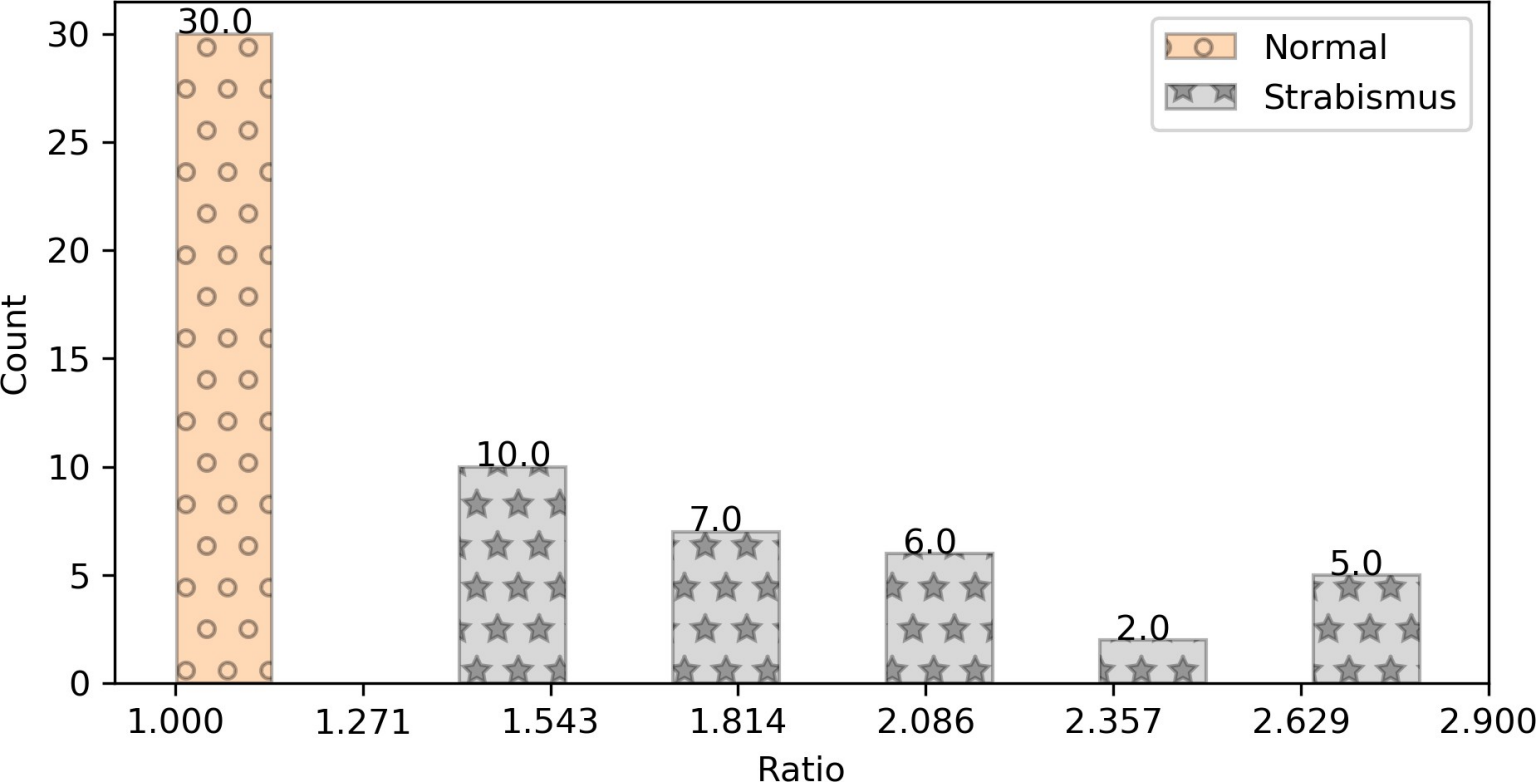
Distribution of positional similarity of normal and strabismus images.

With the pupil center obtained by the LSM and the landmarks detected by the facial landmark detector, we computed the positional similarity of the normal and strabismus images, and the distribution is shown in Fig 8.

The positional similarity estimates of the normal images fall within a small range from 1.002 to 1.139, in which 1.002 was the minimum value of the positional similarity estimate and 1.139 was the maximum estimate. On the other hand, the positional similarity estimates of strabismus images fall within the range from 1.333 to 2.877.

## Discussion

**Fig 9:**
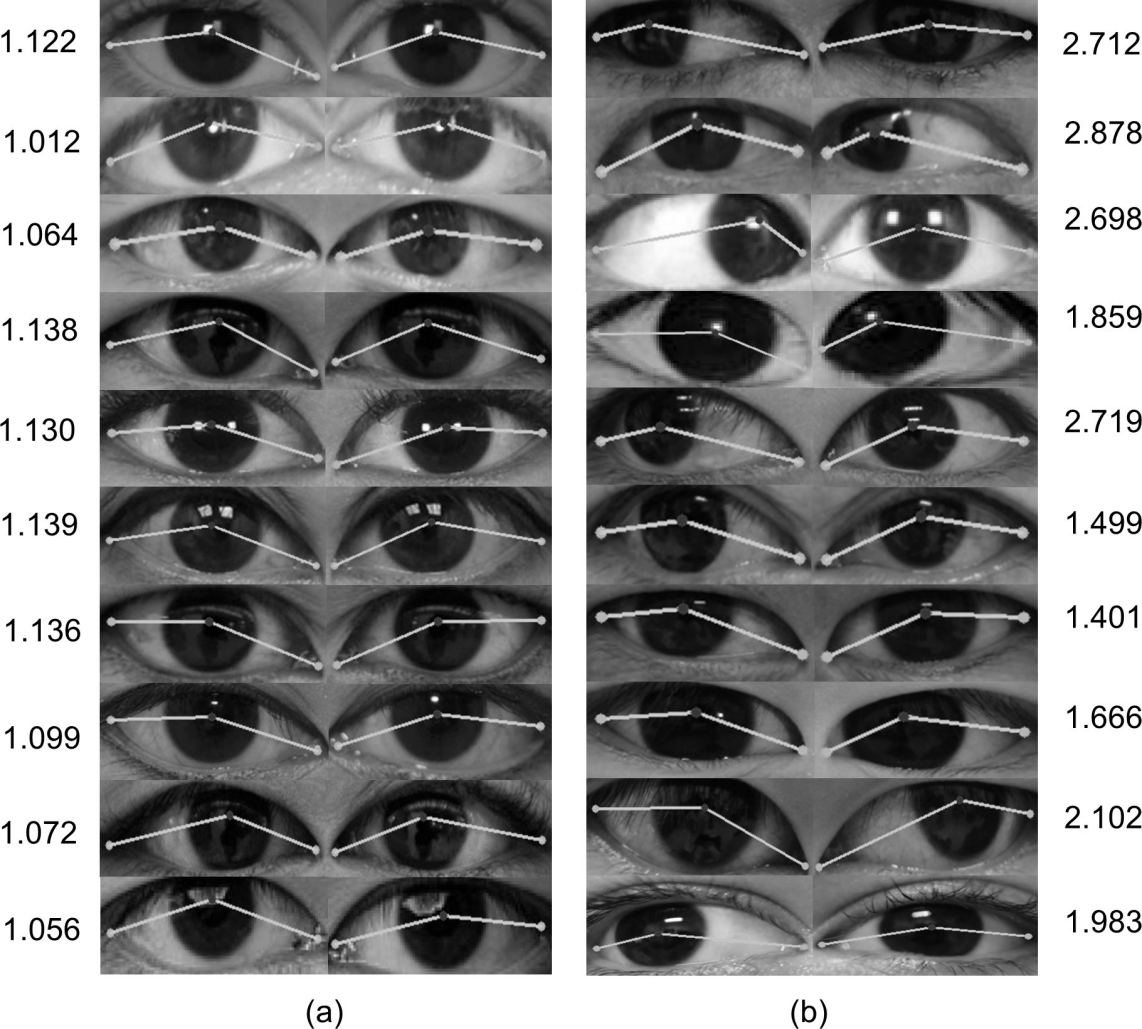
The positional similarity estimates of 10 normal and strabismus images. (a) The normal images. (b) The strabismus images. The numbers on both sides represent the positional similarity estimates.

In the present study, we used the testing images taken under various illumination situations to verify the effectiveness of the proposed method. In the face- and eye-detection stage, the CNN-based model and facial landmark detector successfully detected and extracted the eye regions from the images, which could be attributed to the multiple-feature extractor and accurate classifier trained from a large dataset. Otsu’s binarization method automatically determined an optimal threshold value for the extracted eye region to eliminate the nonrelevant pixel units and retained the iris region. The HSV color model converted the extracted image to the HSV image for separating the iris region from the eyelashes and combined it with the resulting image from Otsu’s binarization method, which made the proposed method more robust in handling the image with different illumination. Therefore, the pixel points located at the iris limbus can be accurately sampled and help to locate the coordinate of the pupil center. By testing the proposed method on normal images, we found that the sample mean and sample standard deviation of the positional similarity estimates were 1.073 ± 0.014 and 0.039, which indicates that the proposed method can efficiently obtain the coordinate of the pupil center and landmarks in estimating the positional similarity. For the results of strabismus images, the sample mean was 1.924 ± 0.169, which was much greater than 1, indicating that the iris position in both eyes was different in strabismus images. Additionally, owing to the existence of diverse types of strabismus in strabismus images as shown in Fig 9, the positional similarity estimates were widely distributed and resulted in a larger sample standard deviation than the one in normal images.

Previous studies on automatic strabismus screening focused on the use of the images featuring traditional screening methods, such as the cover and uncover test and the Hirschberg test, which can only be performed by ophthalmologists. Almeida et al. [16] proposed a strabismus diagnostic system based on the first Purkinje image generated by the Hirschberg test. Almeida et al. [17] further developed a system that can be used for preliminary screening and that aids in the diagnosis of strabismus by using Hirschberg test images obtained at five locations. To obtain the images, the ophthalmologist not only needs to perform the Hirschberg test but must also guide the patient’s sight to the correct position using a ruler and protractor. In another study, Almeida et al. [18] used a digital video featuring a cover test to screen strabismus. Despite the fact that satisfactory detection performances were obtained from the above methods, they require extra labor and on-site image acquisition from the ophthalmologists, which makes them unsuitable when the patients cannot visit the hospitals and undergo the screening tests. On the contrary, the present study focuses on using frontal facial images to screen for strabismus, which does not require any on-site testing and can be easily obtained by non-ophthalmologists (e.g., patients), thereby helping patients to perform strabismus screening without visiting the hospitals. From this aspect, the proposed method can also be an ideal solution for patients who fear visiting hospitals due to the COVID-19 pandemic. Additionally, compared with the above methods, the proposed method could also be extended to measure the ocular deviation by using images obtained via the Hirschberg test. This could be done by applying Otsu’s binarization or a predefined threshold to locate the reflex center and calculating the horizontal and vertical deviation between the reflex center and the pupil center to extract the deviation angle.

With the rapid development of deep learning methods, some automatic strabismus-screening methods based on deep learning have been introduced to reduce the labor burden of traditional methods and help people in remote districts screen for strabismus. Lu et al. [19] presented a strabismus-detection method that includes a CNN architecture for eye region segmentation from the facial image and another CNN architecture for strabismus classification. In another similar study, Zheng et al. [20] employed a pretrained CNN architecture in the classification stage and trained it on the primary gaze photographs. To ensure that the input to the classification network is an eye region image, they performed manual adjustments on the results from the segmentation network. Both works have achieved excellent strabismus-screening performance and demonstrated a capability to automatically screen strabismus via deep learning methods. However, these methods are trained by using datasets of specific populations, and the positional information related to the eyes is not used in the training process, which makes the classification results unpredictable and difficult to interpret when applied to another population. Different from the deep learning-based methods, the present study applied the facial landmark detector to the detected facial region in the segmentation stage to segment the eye region. That is, the eye region was extracted through the surrounding landmarks without an additional manual adjustment. With the extracted eye images, the method located the iris regions and measured their symmetry through the pupil center and the landmarks in order to provide the positional information of two eyes.

Despite the promising results, there are also some limitations of the present study. First, as shown by the mean of the positional similarity estimate of the normal images (slightly greater than 1), the localization of the medial and lateral canthus may not be perfectly accurate due to several possible factors (e.g., skin color, illumination, and inapparent facial contour), which has an impact on the measurement of the positional similarity of two eyes. Second, the upper and lower bound colors of the HSV color model were set to extract the iris region with a color value close to black, which may fail when the iris color is much different from the black color. For enhancing the robustness of the proposed method, the combination of edge-detection and facial landmark model could be a promising solution to provide more accurate localization of the canthi. Additionally, using the HSV color model with an iris color detector could be one solution to increase the generalization ability of HSV. Third, since the proposed method requires images provided by patients living in remote areas for strabismus screening, it may not be suitable for patients who cannot access the internet or do not have digital devices to send images.

In conclusion, the present study provided a convenient and easy-to-use automatic strabismus screening method for patients who live in remote areas and have difficulty accessing eye care centers. The method employs image-processing techniques to measure the positional similarity of two eyes through a frontal facial image that can be easily obtained from a mobile phone or camera. The results shown in the experimental part demonstrate the method could be feasible in allowing patients living in remote areas to take their frontal facial image for strabismus screening. In addition, the measurement of the positional similarity of two eyes could provide ophthalmologists with a priori information about the deviation of a patient’s eyes so they can diagnose strabismus quicker.

## Supporting information

S1 Fig Experimental results of 30 normal and strabismus images. As supporting information, we present experimental results of the total 60 images (30 strabismus, 30 normal), including positional similarity evaluated with the proposed procedure.
(TIF)
