# Supplementary figures and images for: An automatic screening method for strabismus detection based on image processing
Source: PLoS One. 2021 Aug 3;16(8):e0255643. doi: 10.1371/journal.pone.0255643 (PMC8330949; doi:10.1371/journal.pone.0255643)

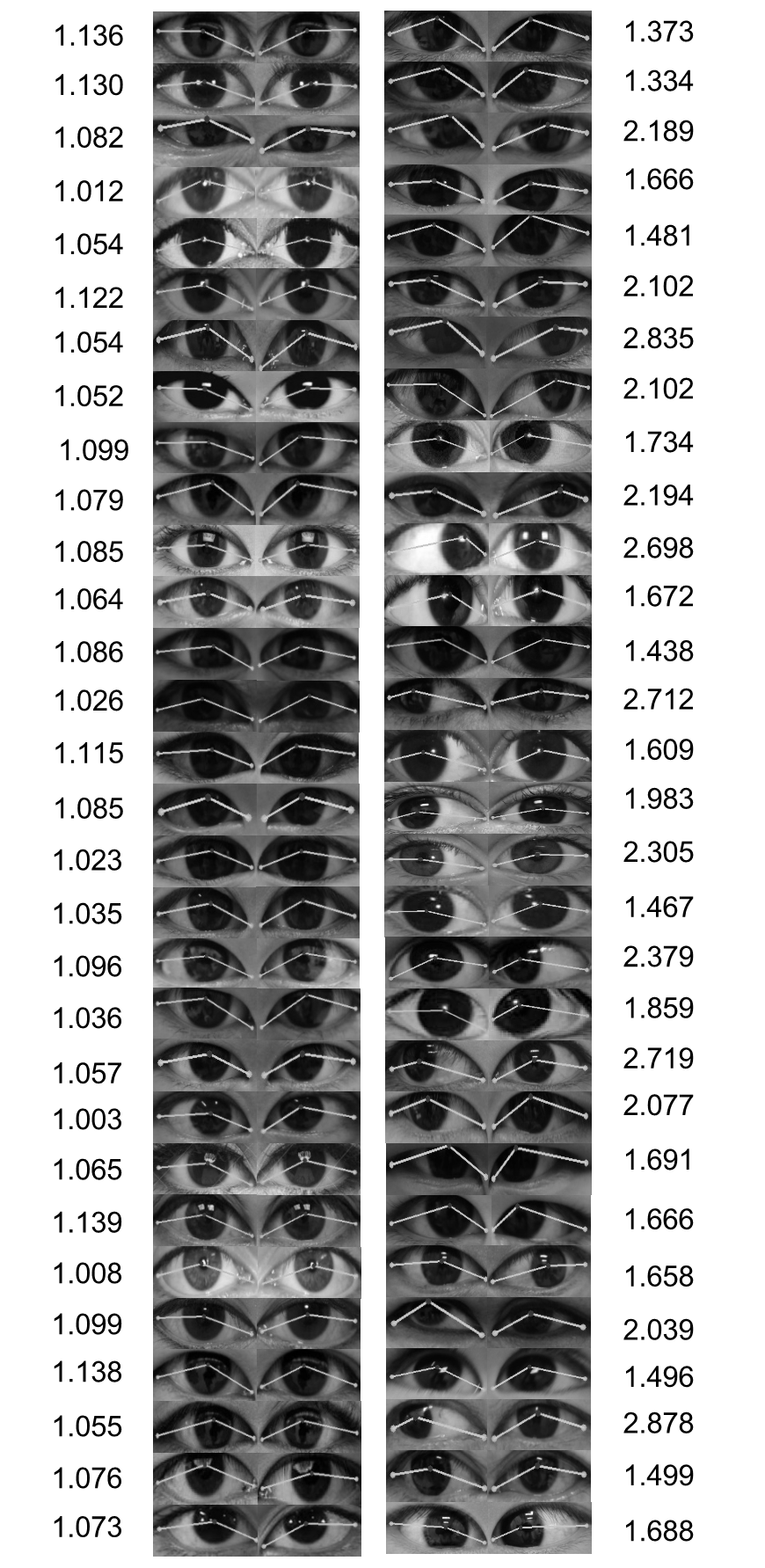

Supplement: S1 Fig — As supporting information, we present experimental results of the total 60 images (30 strabismus, 30 normal), including positional similarity evaluated with the proposed procedure. (TIF) [file pone.0255643.s001.tif]
